# Quality of life in patients with progressive keratoconus treated with corneal collagen crosslinking

**DOI:** 10.1007/s10792-024-03400-6

**Published:** 2025-03-14

**Authors:** J. Steinberg, P. Fischer, A. Frings, V. Druchkiv, T. Katz, S. J. Linke

**Affiliations:** 1 Department of Ophthalmology, University Medical Center Hamburg-Eppendorf, Hamburg, Germany; 2 Medical and Research Center for Ophthalmology, Hamburg Vision Clinic, Hamburg, Germany; 3 Department of Ophthalmology, Heinrich Heine University Düsseldorf, Düsseldorf, Germany; 4 Hamburg Vision Clinic / Zentrumsehstärke, Campus of the University Hospital Hamburg – Eppendorf (UKE), Martinistrasse 64, 20251 Hamburg, Germany

**Keywords:** Keratoconus, Vision-related quality of life, KORQ, NEI-25, Corneal crosslinking

## Abstract

**Purpose:**

This study aims to analyze the vision-related quality of life (vr-QoL) in keratoconus (KC) patients after corneal crosslinking (CXL).

**Methods:**

A prospective clinical study was conducted, wherein 41 patients underwent assessment using the "National Eye Institute Visual Functioning Questionnaire" (NEI-25) and the "Keratoconus Outcomes Research Questionnaire" (KORQ) to evaluate vr-QoL, along with the examination of morphology and functional parameters before CXL, and at three and six months post-treatment. Rasch analyses were used to verify the measurement precision of the KORQ with our study population.

**Results:**

There were no statistically significant changes observed in corneal morphology and best corrected visual acuity after CXL. Analysis of the NEI-25 questionnaires and KORQ demonstrated no statistically significant changes throughout the follow-up period. Rasch analyses revealed a high measurement precision of the KORQ within our population.

**Conclusion:**

The study indicates that patients with progressive KC maintain stable vr-QoL after undergoing CXL. Based on our findings, we suggest considering very early CXL treatment for KC patients that are at high risk of progression.

**Supplementary Information:**

The online version contains supplementary material available at 10.1007/s10792-024-03400-6.

## Introduction

Keratoconus (KC) is a bilateral disease that leads to a decrease in visual acuity and quality due to a progressive irregular ectasia of the cornea. The vision-related changes start during adolescence and tend to stabilize during the 3rd and 4th decades of life [1]. A current meta-analysis by Hashemi et al. reports a worldwide prevalence of 1.38/1000 [2].

Whereas the decrease of visual acuity (VA) during the early stages can be managed with glasses, the increasing irregularities of the cornea during more advanced stages require the use of hard contact lenses and/or surgical interventions, including corneal transplantation, to ensure a VA at least suitable for daily life tasks. In the case where adequate VA is till preserved, corneal collagen crosslinking has been proven to be an effective therapy to stop the further progression of corneal ectasia, thus stabilizing the disease [3–5].

In recent years, numerous studies have been conducted not only to enhance our understanding of the Quality of Life of keratoconus patients but also to improve the methods for its measurement [6–10].

A previous study from our group demonstrated a decline in vr-QoL among patients with progressive KC compared to those with stable disease. In the same study, a subgroup of KC patients undergoing CXL due to progressive disease demonstrated unchanged (decreased) vr-QoL scores 3 months post-treatment [11]. The present study aimed to verify previous findings within a larger cohort over an extended follow-up period of 6 months. Moreover, an additional KC-specific questionnaire was applied to evaluate the vr-QoL.

## Materials and methods

This prospective study was performed as part of a cooperation between the Department of Ophthalmology—University Medical Center Hamburg-Eppendorf—and the Hamburg Vision Clinic, located at the university campus Hamburg-Eppendorf. The study adhered to the principles outlined in the Declaration of Helsinki. We obtained informed consent from all participants and received approval from the local ethics committee. The study is registered in the German Clinical Trials Register (DRKS; DRKS00030330).

The diagnosis of clinically manifest KC in our study was made following a thorough clinical evaluation by two experienced corneal specialists (JS, SL) based on slit-lamp findings and at least one of the following objective topography data: KISA-Index > 100, I-S > 1.4D, Kmax > 47D [12, 13]. The definition of progressive keratoconus included an increase in Kmax and/or refractive astigmatism ≥ 1 D within 1 year [14, 15]. Exclusion criteria comprised previous ocular surgery or trauma, and the use of contact lenses within 5 days (soft CL) or 7 days (hard CL) prior to examination.

To analyze the vr-QoL in our patients, two questionnaires were used: the “National Eye Institute Visual Functioning Questionnaire–25” [NEI-25] and the “Keratoconus Outcomes Research Questionnaire” [KORQ]. Both were evaluated and used in former studies for measuring vr-QoL in patients with keratoconus [6–8, 16, 17]. The questionnaires were completed after one of the authors (PF) informed the patients about the study’s rationale and obtained their written consent. All KC patients, informed about their disease and its progression in one eye beforehand, were scheduled for an epi-off corneal cross-linking treatment (CXL) with regular follow-up examinations at the Hamburg Vision Clinic. Both questionnaires were completed before, three, and six months after CXL.

The changes in refraction, best corrected visual acuity (BSCVA), and topographic parameters were estimated using mixed regression with subject random effect. The differences in estimates between time periods were tested with the Tukey post hoc method. For testing differences of glare-test data, the Wilcoxon signed-rang test was applied. Spearman correlation analyses were used to identify potential correlations between the KORQ results and the measured topographic and VA data.

The data from the KORQ were analyzed using the “ready-to-use Excel spreadsheet” provided and recommended by Khadka et al. [10]. e further evaluated how sufficient the KORQ measures psychometric properties in our patients by performing Rasch analysis. Thereby, the precision can be estimated by testing the one-dimensionality, ordering of categories, and expressed in Rasch outputs for person and item separation and reliability [9, 18]. The NEI-25 data were evaluated as recommended by the Mangione et al. [19].

## Results

In total, 41 patients (33 male, 8 female, mean age: 25 years, range 18–50) with progressive KC were included. Refraction, visual acuity, and topography data for both eyes are displayed in Table 1.

**Table 1:** Descriptivs

	Before surgery			3 months follow-up			6 months follow-up			*P* Value^b^
	N	Range	Mean (SD)^a^	N	Range	Mean (SD)^a^	N	Range	Mean (SD)^a^	
*Treatment-eye*										
ISV (D)	36	− 0.09 to 12.89	6.53 (± 3.63)	31	− 0.9 to 13.4	6.72 (± 3.63)	35	− 0.26 to 11.8	6.51 (± 3.63)	0.410
KMax (D)	36	45.77 to 74.92	56.42 (± 7.46)	31	46.18 to 72.96	56.19 (± 7.45)	35	44.9 to 74.76	55.82 (± 7.45)	0.330
RMS	36	0.33 to 6.33	2.90 (± 1.35)	31	0.58 to 5.96	2.86 (± 1.35)	35	0.67 to 6.12	2.80 (± 1.35)	0.430
Sphere (D)	37	− 10 to 3.5	− 1.15 (± 2.64)	40	− 12 to 4.5	− 0.74 (± 2.64)	39	− 10 to 2.75	− 1.23 (± 2.64)	0.510
Cylinder (D)	37	− 7.75 to 0.25	− 2.89 (± 1.89)	40	− 8 to − 0.5	− 3.48 (± 1.88)	39	− 7.5 to − 0.75	− 3.27 (± 1.87)	0.220
Spherical Equiv. (D)	37	− 10.62 to 2.25	− 2.59 (± 3.15)	40	− 16 to 3	− 2.49 (± 3.16)	39	− 13.5 to 1	− 2.86 (± 3.16)	0.730
UDVA (LogMar)	25	0.15 to 2	0.86 (± 0.40)	33	0.15 to 2	0.75 (± 0.33)	30	0.15 to 2	0.66 (± 0.29)	**0.010**
CDVA (LogMar)	36	0 to 2	0.28 (± 0.17)	40	0 to 1	0.26 (± 0.17)	39	0 to 0.82	0.26 (± 0.17)	0.560
*Fellow eye*										
ISV	36	− 1.8 to 20.83	4.33 (± 3.97)	31	− 0.32 to 20.47	4.67 (± 3.97)	34	− 9.46 to 11.01	3.51 (± 4.07)	0.140
KMax	36	41.62 to 94.52	52.70 (± 9.81)	31	41.98 to 84.94	52.56 (± 9.88)	34	42.03 to 89.57	52.12 (± 9.81)	0.720
RMS	36	0.23 to 7.08	2.10 (± 1.63)	31	0.33 to 7.14	2.11 (± 1.62)	34	0.26 to 15.55	2.37 (± 1.64)	0.600
Sphere (D)	37	− 14.5 to 8	− 0.58 (± 2.79)	39	− 13 to 4.75	− 0.53 (± 2.79)	38	− 11.5 to 8	− 0.23 (± 2.80)	0.460
Cylinder (D)	37	− 5 to 0	− 1.44 (± 1.30)	39	−	−	38	− 6 to 0	− 1.75 (± 1.30)	0.130
Spherical Equiv. (D)	37	− 16.25 to 6.25	− 1.28 (± 2.90)	39	− 15.5 to 3.38	− 1.26 (± 2.90)	38	− 14.25 to 7	− 1.10 (± 2.91)	0.850
UDVA (LogMar)	25	0 to 2.3	0.54 (± 0.30)	31	0 to 2	0.48 (± 0.28)	30	0 to 2	0.41 (± 0.16)	**0.040**
CDVA (LogMar)	36	0 to 2.3	0.13 (± 0.12)	39	0 to 2	0.13 (± 0.13)	38	0 to 2	0.12 (± 0.11)	0.490

^a^Estimated with mixed regression model with subject random effect

^b^Adjusted with Tukey method

Among the refractive, topographic, and visual acuity data, we identified statistically significant differences only for uncorrected distance visual acuity (UDVA) in both the treated eye and the fellow eye. The pairwise comparison revealed statistically significant differences only between pre-treatment and six-month post-treatment measurements, with improvement in both eyes (treated eye: mean UDVA (LogMar) before/after treatment: 0.86/0.66, *P* = 0.009; fellow-eye: 0.66/0.45, *P* = 0.029). We also conducted contrast and glare VA test (Mesotest®, Oculus, Wetzlar Germany) with all patients who failed to identify any optotypes either before or after the treatment.

### Psychometric analysis

#### KORQ

The KORQ and the NEI-25 were completed on the day of surgery, three months (mean/SD: 93 days ± 13), and six months (189 days ± 15) after CXL.

During the follow-up period, the psychometric properties data obtained from the KORQ were analyzed. We found statistically significant differences fo ronly one of the 18 items in the “visual ability” section: “How much does your vision interfere with using a computer screen?” (improvement from pre- to 6-month follow-up, *P* = 0.033). Within the “activity” section, two of the 11 items demonstrated statistically significant differences: “How much are you troubled by distorted vision?” (improvement from pre- to 6-month follow-up, *P* = 0.004) and “Do you experience any discomfort or irritation due to dry eyes on dry days?” (decline from pre- to 6-month follow-up, *P* = 0.015). However, the combined scores for both activity and symptoms did not show any statistically significant differences (activity [pre/3 month post/ 6 months post]: − 1.18(+  − 0.98)/ − 1.23(+  − 0.99)/ − 1.27(+  − 1.04), *P* = 0.799; symptoms: − 1.25(+  − 0.98)/ − 1.3(+  − 0.93)/− 1.24(+  − 1.01), *P* = 0.738, for further data see supplement file 1). We did not find any statistically significant correlations between either “symptoms” or “activity” scores and the analyzed topographic or visual acuity data before or after the treatment.

To evaluate the precision of the KORQ in our study population, we additionally conducted Rasch analyses. The output numbers for our study collective (Table 2) demonstrate a high measurement precision (the cut-offs displayed in Table 2 are based on the study by Khadka et al. [10]).

**Table 2:** Rasch analysis results to evaluate the psychometric properties of the KORQ in our study population

Categories	Domains	
	Symptoms	Activity
Person separation (> 2.0)	2.07	2.83
Person reliability (> 0.8)	0.81	0.89
Item separation (> 2.0)	3.03	7.15
Item reliability (> 0.8)	0.9	0.98

#### NEI-25

Analyzing potential differences between the vr-QoL of KC patients with the NEI-25, we did not find statistically significant differences in items before and after the treatment (see supplement file 2).

## Discussion

In the present prospective study, we corroborate previous findings [11] from our group and observe similar results in a larger cohort and over an extended follow-up period. This indicates a stable vr-QoL in KC patients following corneal crosslinking. These results align with the findings of other studies using the NEI-25 to assess vr-QoL in progressive KC post-CXL [20, 21]. Kandel et al. published a comprehensive review on measuring QoL in KC, emphasizing the importance of using a detailed and high-quality patient-reported outcome measure to obtain reliable results.

The NEI-25 was designed in 2001 to measure the dimensions of self-reported vision-targeted health status that are most important for persons who have chronic eye diseases. The content was identified during a series of condition-specific focus groups with patients who had age-related cataracts, glaucoma, age-related macular degeneration, diabetic retinopathy, or CMV retinitis [19, 22]. Besides the content-related inaccuracies when using a questionnaire originally designed to analyze patients with different pathologies, the method of designing the questionnaire is crucial for achieving reliable results. Several questionnaires, like the NEI-25, use traditional Likert (summary) scoring without the application of statistical validation derived from item response theory [23]. Important components of these statistical validation options include Rasch weighting, converting standard Likert scales into linear data and removing redundant items, or principal component analysis, establishing whether items on a scale all measure the same trait, or whether a scale is unidimensional [24, 25]. In 2017, based on these principles (importance of pathological specific content and statistical validation implementation), Khadka et al. developed a KORQ, which was supposed to represent “the only validated keratoconus-specific questionnaire”, providing “most superior psychometric properties” [8, 10]. Consequently, in more recent studies, the KORQ has been predominantly used to analyze vr-QoL in KC patients [6, 7, 26, 27].

To strengthen the reliability of our findings, we complemented the NEI-25 by using the KORQ and additional Rasch analyses to validate the precision of the KORQ when we applied them to our study population. Although individual items of the KORQ display statistically significant changes following CXL (3 of 29, with 2 items showing improvement of VA-related tasks and one item showing an increase of dry eye symptoms), neither the combined “activity” nor the “symptoms” scores demonstrated differences of statistical significance. The Rasch analysis revealed good precision using the KORQ in our population.

Two previous studies (Kandel et al. and Ferrini et al.) analyzed the vr-QoL in KC patients pre and post-CXL using the KORQ, proving either improvement or no changes in vr-QoL [6, 7]. These studies, similar to our study, included 39 or 38 patients with a follow-up of 6 months. However, their study found a correlation between visua and morphology parameters with the KORQ scores. This finding was completely unexpected, as most studies typically report a high correlation between vr-QoL scores and visual acuity [6, 7, 10]. Examing the data given in the respective studies, the most obvious biasing factors leading to different correlation results might be a distinct heterogeneity of the age and the VA data. The mean age of the patients from the study made by Khadka et al. was 45 years of age, which is almost double the mean age of the patients included in our study, and in the studies by Ferrini et al. (20.5) and Kandel et al. (24,2). Regarding the influence of the visual acuity data, Ferrini et al. reported a mean BCVA of the treated eye of 0.2 before the treatment without giving information about the BCVA of the fellow eye. Their correlation analysis, which was conducted for both eyes of the patients, revealed significant correlations of the KORQ scores and the treated eyes before, but not at the 6-month follow-up. The untreated eyes showed no significant correlations between the BCVA and the KORQ scores for “symptoms” or “activity”. Kandel et al. report a mean BCVA of the treatment eye of 0.4 before the treatment, without presenting data on the untreated eye. The initial BCVA of 0.4 is much lower than that in similar studies. Khadka et al. analyzed patients with KC but did not assess them before and after CXL. Their study reported on the ‘worse’ and ‘better’ eyes, using the ‘‘better eye’’ for statistical analysis. The BCVA was 0.2 for the better eyes and 0.3 for the worse eyes.

Despite the variability in BCVA reported across different studies, there is still no clear consensus on which eye should be used for correlation analysis—whether it should be the treated eye, the ‘‘better’’ eye, or the binocular visual acuity. This lack of clarity complicates the interpretation and comparison of the results across all studies.

A recent study by Kandel et al. aimed to gain deeper insights into the relationship between QoL scores and standard clinical variables. The study reported that the correlations of QoL scores with most clinical parameters and visual acuity were similar, regardless of whether the better or worse eye was considered [28]. They also demonstrated statistically significant correlations between clinical and QoL scores, but only with “low magnitudes” suggesting a more complex relationship between clinical parameters and patient-reported outcomes [28].

A potential drawback of our study could be the heterogeneity of the VA among the included patients. Usually, we perform CXL in patients with a “still good enough vision for daily tasks”. Otherwise, we use a combined approach (PRK/CXL), utilize implant rings (MyoRing®), or resort to keratoplasty. However, some patients did not follow our recommendation and decided on the “more basic” (CXL) treatment which is often the only treatment option fully covered by their insurance company. On one hand, we could have chosen to exclude these patients; however, we intentionally included all patients treated in our facilities to avoid unrecognized bias. This approach allowed us to gain an "unfiltered" insight into the "general" vr-QoL of our KC patients.

## Conclusions

In summary, the present study confirms our previous findings of a stable (not improving) vr-QoL after CXL in progressive KC patients. These findings are particularly important considering the fact of a known lower vr-QoL in KC patients with a diagnosed progression compared to those with stable disease [11, 29]. Our findings support the importance of early stabilizing treatment for KC patients, not only from a morphological or functional aspect but also from a psychological perspective [11]. Based on this understanding, we recommend considering initial stabilizing treatment for young patients at a high risk of progression, rather than waiting until progression can be confirmed by morphological changes.

## Supplementary Information

Below is the link to the electronic supplementary material.

Supplementary file1 (XLSX 14 KB)

Supplementary file2 (XLSX 13 KB)
